# Modulation of ligand–heme reactivity by binding pocket residues demonstrated in cytochrome c' over the femtosecond–second temporal range

**DOI:** 10.1111/febs.12526

**Published:** 2013-10-11

**Authors:** Henry J. Russell, Samantha J. O. Hardman, Derren J. Heyes, Michael A. Hough, Gregory M. Greetham, Michael Towrie, Sam Hay, Nigel S. Scrutton

**Affiliations:** ^1^ Faculty of Life SciencesManchester Institute of Biotechnology and Photon Science InstituteThe University of ManchesterUK; ^2^ Central Laser FacilityResearch Complex at HarwellDidcotUK; ^3^ School of Biological SciencesUniversity of EssexUK

**Keywords:** cytochrome c', nitric oxide binding, protein dynamics, time‐resolved infrared, transient absorption

## Abstract

The ability of hemoproteins to discriminate between diatomic molecules, and the subsequent affinity for their chosen ligand, is fundamental to the existence of life. These processes are often controlled by precise structural arrangements in proteins, with heme pocket residues driving reactivity and specificity. One such protein is cytochrome c', which has the ability to bind nitric oxide (NO) and carbon monoxide (CO) on opposite faces of the heme, a property that is shared with soluble guanylate cycle. Like soluble guanylate cyclase, cytochrome c' also excludes O_2_ completely from the binding pocket. Previous studies have shown that the NO binding mechanism is regulated by a proximal arginine residue (R124) and a distal leucine residue (L16). Here, we have investigated the roles of these residues in maintaining the affinity for NO in the heme binding environment by using various time‐resolved spectroscopy techniques that span the entire femtosecond–second temporal range in the UV‐vis spectrum, and the femtosecond–nanosecond range by IR spectroscopy. Our findings indicate that the tightly regulated NO rebinding events following excitation in wild‐type cytochrome c' are affected in the R124A variant. In the R124A variant, vibrational and electronic changes extend continuously across all time scales (from fs–s), in contrast to wild‐type cytochrome c' and the L16A variant. Based on these findings, we propose a NO (re)binding mechanism for the R124A variant of cytochrome c' that is distinct from that in wild‐type cytochrome c'. In the wider context, these findings emphasize the importance of heme pocket architecture in maintaining the reactivity of hemoproteins towards their chosen ligand, and demonstrate the power of spectroscopic probes spanning a wide temporal range.

**Abbreviations**
*Ax*Cytcp: *Alcaligenes xylosoxidans* cytochrome c'
CO: carbon monoxide
NO: nitric oxide
TA: transient absorption
TRIR: time‐resolved infrared
WT: wild‐type

## Introduction

**Scheme 1:**
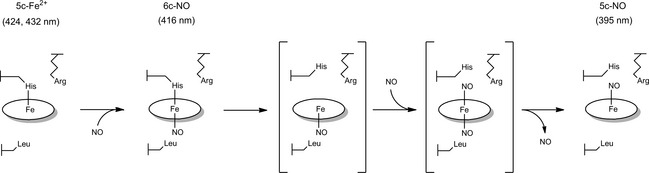
The currently proposed distal‐to‐proximal mechanism of NO binding in A*x*Cytcp (adapted from 11). Initially, an NO molecule binds to the hydrophobic distal face forming a 6c‐NO species. The Fe–His bond is then cleaved, allowing a second NO to bind to the proximal face, forming a putative dinitrosyl intermediate. Finally, the distal NO detaches, leaving a proximally bound 5c‐NO species. Hypothesized, short‐lived intermediates are indicated by square brackets. Arg124 is thought to prolong the lifetime of the 6c‐NO distal species by steric and/or electrostatic effects on His120.

**Figure 1:**
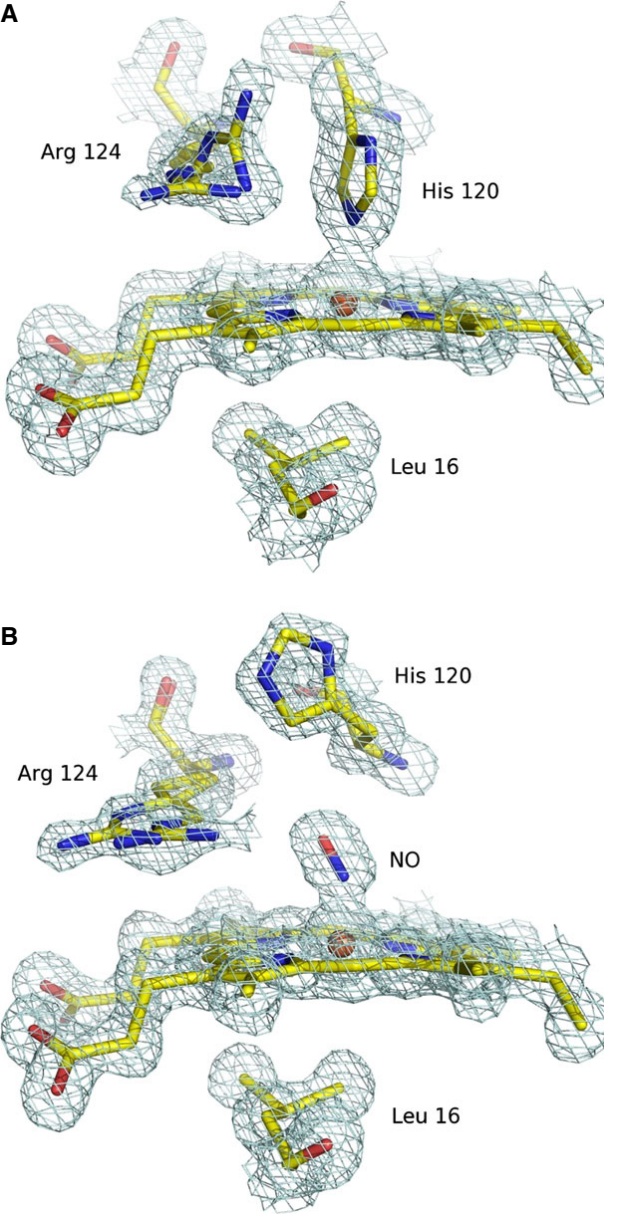
The heme environment in crystal structures of (A) ferrous A*x*Cytcp at 1.45 Å resolution (PDB ID 2YLI) 12 and (B) ferrous, NO‐bound A*x*Cytcp at 1.2 Å resolution (PDB ID 2XLM) 13, with key binding‐pocket residues shown.

Due to their fundamental roles in biochemical pathways as widespread as respiration, vasodilation and drug metabolism 1, hemoproteins continue to attract major interest in relation to mechanisms of diatomic gas binding. Discrimination between nitric oxide (NO), carbon monoxide (CO) and molecular oxygen (O_2_) by hemoproteins is a remarkable example of biological specificity, and one of the most challenging questions at present is understanding how the protein structure regulates the affinity of the heme cofactor towards these diatomic gases 2. Hemoproteins are known to select between these similar diatomic gases in order to modulate their functionality, for example as sensory proteins or gas transporters. Mono‐His cytochrome c' (Cytcp) is present in a variety of nitrogen‐fixing, denitrifying and photosynthetic bacteria. Physiologically, Cytcp has been implicated in NO transport and reduction of intracellular NO toxicity 3. In a remarkable example of ligand discrimination and specificity, Cytcp excludes O_2_ completely from the heme binding site, but it binds CO on the distal face forming a 6‐coordinate heme, and binds NO on the proximal face as a 5‐coordinate heme 7. The proximal binding of NO as a 5‐coordinate adduct is highly unusual. However, a protein that has been hypothesized to share this feature is soluble guanylate cyclase, an NO sensor that facilitates vasodilation and neurotransmission 1. Previous studies have suggested that soluble guanylate cyclase binds NO in a two‐step biomolecular mechanism that yields a proximally bound 5c‐NO complex 9. As no structural data are available for the heme domain of soluble guanylate cyclase, mechanistic understanding of soluble guanylate cyclase activation by NO has benefitted greatly from studies of Cytcp 9. The crystal structures of *Alcaligenes xylosoxidans* cytochrome c' (*Ax*Cytcp) in the ferrous and NO‐bound forms are shown in Fig. 1 11, highlighting the 5c proximally bound histidine (Fig. 1A) and the heme‐bound NO (Fig. 1B). A proposed NO binding mechanism is outlined in Scheme 1, based on a number of spectroscopic and crystallographic studies 11. Initially, NO binds distally to form a 6c‐NO intermediate, weakening the proximal His–Fe bond due to the repulsive *trans* effect of NO binding. This allows displacement of the His residue from the heme by a second NO molecule, forming a putative dinitrosyl intermediate, from which the distal NO dissociates to leave the proximal 5c‐NO adduct.

**Figure 2:**
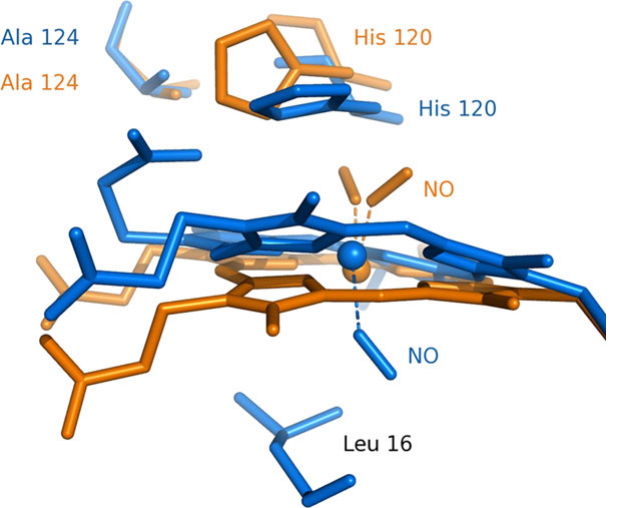
The heme environment for NO‐ligated R124A shows a mixture of 5c‐NO species. The major conformation (orange) is a proximally bound 5c‐NO species with two orientations of the bound ligand (occupancies of 0.3 and 0.4), while the minor conformation (blue) has a distally bound 5c‐NO with the heme face shifted into the cavity vacated by the proximal arginine residue (0.3 occupancy). PDB IB 2XL6, adapted from 13.

Studies involving an L16A variant of *Ax*Cytcp have suggested that the hydrophobic distal pocket (including L16) is crucial to the NO binding mechanism and ligand discrimination 12. With the L16A variant, NO binds distally as a 6c‐NO adduct, but does not disrupt the His–Fe bond, causing this distal complex to be trapped. Furthermore, in this variant, CO has been observed to bind with the highest affinity reported for any hemoprotein 12, and O_2_ is no longer excluded from the distal face 17. Another residue that contributes to the NO binding mechanism is Arg124 in the proximal heme pocket. When this residue was exchanged for an alanine residue (R124A), the His–Fe–NO intermediate was not detected spectroscopically, suggesting that R124 plays a part in extending the lifetime of this intermediate 13. Also, the crystal structure of this variant revealed a mixture of proximally bound 5c‐NO (analogous to the wild‐type) and distally bound 5c‐NO with the heme shifted ‘up’ into the cavity vacated by Arg124 (Fig. 2) 13. These findings suggest that the stability of heme binding to the protein scaffold is somehow facilitated by R124, and emphasize its importance structurally.

Transient absorption (TA) and resonance Raman measurements have shown that wild‐type *Ax*Cytcp (WT) binds 5c‐NO in a highly controlled environment 18. This is demonstrated by the high proportion of geminate recombination after excitation (~ 99%) on an ultrafast time scale (τ = 7 ps) 18. Laser‐flash photolysis studies have shown that only ~ 1% of the population releases NO to the surrounding bulk solvent, with rebinding of the proximal histidine to the heme Fe 20. This re‐formation of the His–Fe bond prevents NO rebinding on the proximal face and has been described as a ‘kinetic trap’ mechanism. Any further NO rebinding cannot occur directly to the proximal face but must occur via the 6c distal intermediate as the 6c distal intermediate enables Fe–His bond breakage. These findings highlight the role of the proximal pocket structure in minimizing NO escape, with the aforementioned proximal arginine (R124) hypothesized to directly protect against escape to the surrounding solvent 19.

As the control of NO binding is thought to be diminished in the R124A and L16A variants, we have investigated the roles of these residues in controlling the heme pocket environment with respect to NO binding in *Ax*Cytcp. We used pump‐probe TA experiments and laser‐flash photolysis to continuously sample all relevant time scales from femtoseconds to seconds for NO rebinding. Furthermore, these TA studies have been complemented with time‐resolved infrared (TRIR) measurements in the femtosecond to nanosecond range. TRIR is a powerful tool for analyzing molecular vibrations following photoexcitation, providing direct information on bond cleavage and re‐formation, as well as any protein–heme interactions. Thus, the geminate recombination events observed following photoexcitation in the femtosecond to nanosecond range may be probed by TA (monitoring electronic transitions) and TRIR (monitoring vibrational transitions). For R124A in particular, these experiments have revealed the crucial role for this residue in modulating heme pocket reactivity for NO rebinding and control of NO escape to the bulk solvent.

## Results and Discussion

### Wild‐type *Ax*Cytcp

**Figure 3:**
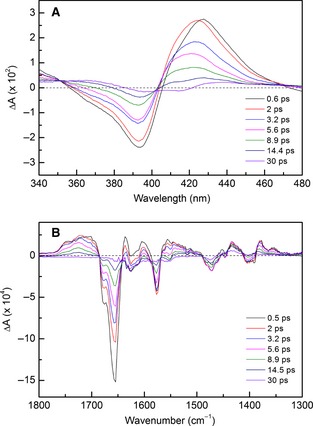
TA (A) and TRIR (B) difference spectra relative to the ground state for WT following photolysis of the bound NO by laser excitation. TA difference spectra from 0.5 to 30 ps illustrate a ground‐state bleach at 393 nm and an equivalent transient at 420 nm that return to the ground state almost completely after 30 ps. The most noteworthy feature in the TRIR difference spectra is the 1656 cm^−1^ signal bleach corresponding to cleavage of the 5c‐NO bond.

Initially TA and TRIR analysis of WT was performed in order to compare our findings against existing data, and also to provide previously unreported vibrational spectroscopy data. For both TA and TRIR experiments, a similar excitation wavelength (532 nm), laser power (1 μJ) and beam diameter (~ 150 μm) were used, so the spectral and kinetic data may be directly compared. Furthermore, these experimental conditions were also used in previous ultrafast studies of WT 18, allowing additional comparative analysis. The TA difference spectra are shown in Fig. 3A for selected data between 0.5 and 30 ps, illustrating the same spectral features as previously observed 18. These include a ground‐state bleach at 393 nm and an equivalent transient feature centered at 420 nm, both of which decay almost completely to the ground state over ~ 100 ps (see Fig. S1 for ground‐state UV‐vis absorption spectra of WT and variants). The minimal signal remaining after 100 ps (~ 5% of the initial signal remaining at 396 nm) has previously been assigned to the 5c‐His species formed upon NO escape to the surrounding solvent 19.

Figure 3B illustrates TRIR difference spectra at selected time intervals after excitation equivalent to the TA spectra in Fig. 3A (see Fig. S2 for ground‐state IR absorption spectra of WT and variants). A number of features are evident in this spectral region, the most prominent of which include signal bleaches at 1656, 1678 (shoulder) and 1577 cm^−1^, and transient features at 1717, 1745 (shoulder), 1634, 1602 and 1556 cm^−1^. There are also a number of less pronounced features from 1300 to 1500 cm^−1^. The 1656 cm^−1^ bleach has previously been assigned to the 5c‐NO species 15, and therefore provides the opportunity to directly monitor NO geminate recombination. The overall TRIR difference spectra suggest that the vast majority of photolyzed NO in WT undergoes geminate recombination over a fast time scale, as previously suggested 18.

In order to determine which spectral features correspond to heme vibrations following excitation, control TRIR experiments were performed using reduced WT, R124A and L16A in the absence of NO. As expected, the difference spectrum for reduced WT exhibits a markedly reduced 1656 cm^−1^ band, but retains a number of other spectral features (Fig. S3). When compared directly (Fig. S4), the TRIR data for WT in the presence and absence of NO have a number of common spectral features, particularly in the fingerprint region of 1300–1500 cm^−1^. We have therefore assigned these signals below 1600 cm^−1^ to excitation of the heme and structural changes in coordinating protein residues, including the covalently linked Cys116 and Cys119 21. It is noteworthy that the 1573 cm^−1^ band has been previously suggested to report on either the histidine or arginine residue during NO binding 15. Our data suggest that this is improbable given that this signal bleach is present in the WT with NO bound (histidine displaced), in addition to reduced samples of WT, R124A and L16A (histidine attached, and, in the case of R124A, arginine absent; Fig. S5).

Major TRIR features between 1600 and 1800 cm^−1^ (other than the assigned 1656 cm^−1^ 5c‐NO bleach) include transient absorption features at 1634 and 1717 cm^−1^. The 1717 cm^−1^ band may correspond to the emergence of carbonyl (C=O) groups following excitation, as these groups absorb strongly between 1670 and 1820 cm^−1^ due to a stretching motion 22. According to the WT NO‐bound crystal structure 13, His120 is hydrogen‐bonded to an aspartate residue (Asp121) in contrast with the reduced structure. Therefore, following excitation of NO, this His–Asp hydrogen bond may potentially cleave, allowing competition between the His and NO for proximal binding, and temporarily reveal the carbonyl group of Asp121. If this hypothesis is correct, it suggests that the Asp–His hydrogen bond is immediately cleaved upon NO–heme photolysis. However, this band is particularly broad for a single carbonyl stretch, and the possibility that other vibrational features contribute has not been ruled out. The cleavage of the Asp–His hydrogen bond also offers an explanation for the transient feature at 1634 cm^−1^, as histidine has been previously shown to absorb IR at approximately this frequency 23. A summary of our TRIR assignments for WT following excitation is shown in Table S1.

Kinetics for the TA and TRIR spectra were calculated by globally fitting the signal decays at five distinct wavelength or wavenumber values, using shared lifetimes and a non‐linear least‐squares fitting model (WT global fitting, shown in Figs S6 and S7). This returned three spectral components compared with the two previously reported (Table 1) 19; the previously observed ~ 7 ps component, which corresponds to geminate recombination of NO, and the ~ 100 ps component corresponding to His rebinding are also observed in our data. The variation in τ_3_ between the TA experiments is probably due to the low amplitude of this lifetime component (with the consequent large error). The TRIR experiments were used principally to determine vibrational changes during geminate recombination (initial 10 ps after excitation), and therefore very few data points at longer times were acquired, which explains the poorly resolved τ_3_ value for these data. As the 1656 cm^−1^ band of the TRIR has been previously assigned to the Fe–NO bond 15, this allows the geminate rebinding of NO to be directly monitored. This is particularly beneficial in determining the identity of the previously unresolved fast component (τ_1_). For the majority of NO‐binding hemoproteins, geminate recombination occurs mono‐exponentially over ~ 7 ps 24, with any faster components being due to vibrational relaxation of the heme after laser excitation 25. However, the NO signal bleach in the TRIR (1656 cm^−1^) suggests that this fast component also corresponds to NO rebinding, as the signal amplitude is returning to the ground state, as shown in Fig. S8. The first solved crystal structure of WT suggested that multiple conformers of the NO–heme complex exist, and therefore this component may represent fast recombination of a proportion of NO in a different conformation 11. However, this hypothesis must be treated with caution as a later crystal structure indicated only one orientation of bound NO 13. It is noteworthy that this fast component is unlikely to correspond to vibrationally excited NO rebinding to the heme, as no obvious bandshifts are observed, particularly of the 1656 cm^−1^ feature 28. The 1717 cm^−1^ feature, which we earlier assigned to Asp121 in the heme pocket, fits to a single exponential with τ_1_ = 8.15 ± 0.21 ps, which approximately corresponds to the rate of NO geminate recombination. Therefore, it remains possible that this Asp121 residue gives rise to IR absorbance until NO undergoes geminate recombination and the Asp–His hydrogen bond is re‐formed.

**Table 1:** Kinetic lifetimes of WT from TA and TRIR data (Fig. 3A,B, respectively) when globally fitted to the sum of three exponentials. These lifetime values were generated by global fitting to signal decays at the following frequencies: 377, 393, 417, 428 and 440 nm for TA data, and 1577, 1596, 1625, 1637 and 1655 cm^−1^ for TRIR data. The τ_3_ value in the TRIR was poorly resolved due to the low number of data points acquired beyond 20 ps. These values are compared to existing kinetic data 19

	TA (ps)	TRIR (ps)	Literature TA (ps) 19
τ_1_	2.19 ± 0.18	1.31 ± 0.16	–
τ_2_	5.65 ± 0.15	6.13 ± 0.29	7 ± 0.5
τ_3_	204 ± 56	> 20	100 ± 10

Additional laser photoexcitation experiments over longer time scales revealed no further spectral changes on the ns–μs time scale for WT. Hence, the geminate recombination of NO to WT is complete on the fs–ns time scale, suggesting a highly controlled NO rebinding mechanism following photolysis, consistent with the crowded nature of the proximal pocket. However, laser flash photolysis data (μs–s) show that the remaining 5% of signal amplitude in the TA measurements represents NO escape from the heme binding pocket, which subsequently rebinds from the bulk solvent on a much slower time scale (Fig. S9). The increase in absorbance at 396 nm, which represents formation of 5c‐NO species, may be fitted to the sum of two exponentials with lifetimes of 2.7 ± 0.1 and 76.7 ± 0.2 ms. If one assumes saturating NO concentrations in solution (2 mm), these lifetime values roughly equate to reported *k*_on_ rates for the distal‐to‐proximal binding mechanism from stopped‐flow experiments (literature values of 4.4 × 10^4^ and 8.1 × 10^3^ m^−1^ s^−1^; flash‐photolysis values of 1.85 × 10^5^ and 6.5 × 10^3^ m^−1^ s^−1^) 16.

### L16A *Ax*Cytcp

**Figure 4:**
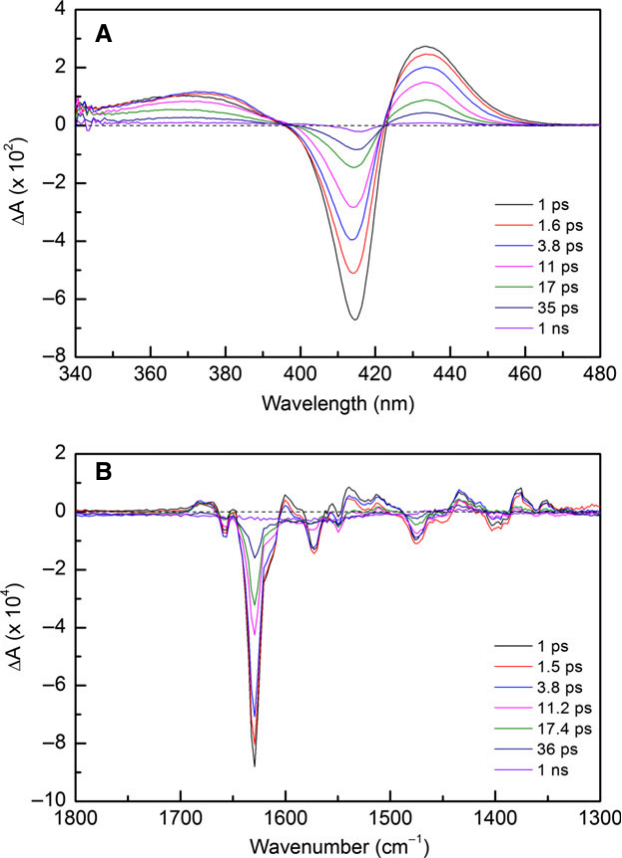
L16A TA (A) and TRIR (B) difference spectra at equivalent time points following excitation between 1 ps and 1 ns. TA difference spectra exhibit a ground‐state bleach at 416 nm, in accordance with the ground‐state spectrum, and transients at 370 and 435 nm. The TRIR shows a signal bleach at 1629 cm^−1^ which probably corresponds to loss of a 6c‐NO species, but the majority of other spectral features are also present in the WT TRIR spectra.

Previous studies have shown that L16A binds NO on the distal face in a 6c‐NO mode (with the proximal histidine still attached) 12, without proceeding to a proximal binding mode. Consequently, this variant of cytochrome c' is a useful comparative system. The TA and TRIR difference spectra for L16A are illustrated in Fig. 4, both of which are distinct from the WT spectra. The TA difference spectra exhibits a ground‐state bleach centered at 416 nm, which corresponds to the ground‐state UV‐vis absorption spectrum of 6c‐NO (Fig. S1), and transient features at 370 and 435 nm that decay to the ground state within 1 ns. The TRIR data show obvious spectral differences compared with WT (Fig. S10), with the ground‐state bleach shifted from 1656 to 1629 cm^−1^. This is in agreement with an earlier assignment of a 6c‐NO species at this position 15, and therefore probably represents the release of NO from the 6c distal binding site, presumably leaving a 5c‐His intermediate. The broad transient feature at 1717 cm^−1^ that was present in the WT sample is missing in the L16A sample. This complements our hypothesis stating that the > 1700 cm^−1^ features report on the Asp121 carbonyl group, as this residue is no longer hydrogen‐bonded to His120 when NO is bound in the 6c distal position. It appears from the TRIR difference spectra that the ground‐state molecule is fully re‐formed after ~ 1 ns, with no escape of NO to the bulk solvent, and this is confirmed by the lack of any signal on the μs–s time scales. This is not a surprising finding as any NO released from the distal pocket into the solvent may simply rebind in the absence of any kinetic trap mechanism (as proposed with WT). This is reflected in the kinetics generated from global fitting of the TA and TRIR data, which fitted to the sum of two exponentials. As with WT, this may correspond to two conformations of NO binding distally, but, in the absence of an L16A crystal structure, this cannot be confirmed. In addition, the lifetime values for L16A appeared to be NO concentration‐dependent, which represents an unusual feature when considering geminate recombination processes. Due to these anomalous findings, the L16A TA and TRIR data have been used for qualitative comparison with WT and R124A, rather than quantitative comparison. The L16A lifetime values are provided in Table S2.

### R124A *Ax*Cytcp

**Figure 5:**
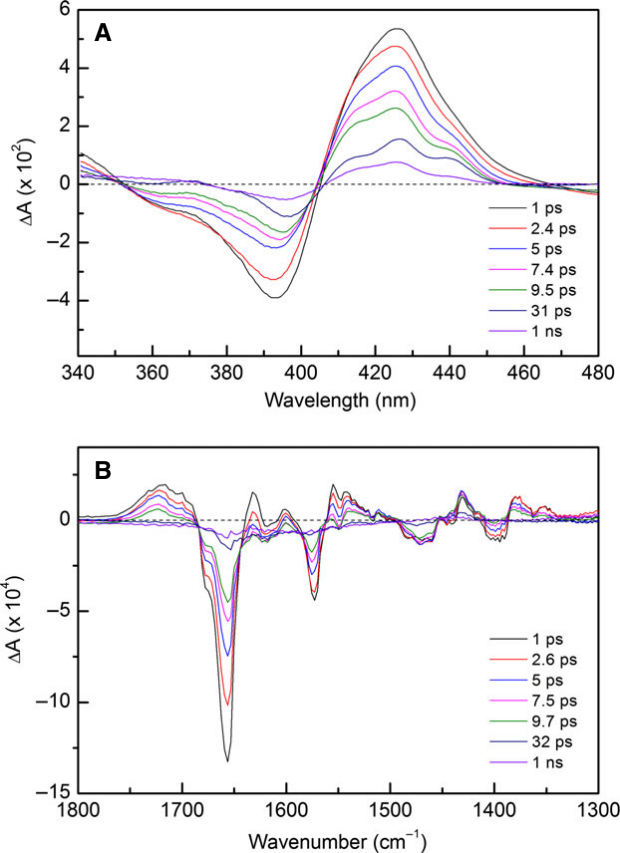
R124A TA (A) and TRIR (B) at selected time points between 1 ps and 1 ns. The TA difference spectra exhibit clear differences from those for WT, with transient features at 414, 426 and 440 nm and an enhanced spectral profile at 1 ns. In contrast, TRIR difference spectra exhibit broadly similar spectral features to WT.

The TA and TRIR difference spectra for the R124A variant at selected time points between 1 ps and 1 ns are shown in Fig. 5. The TA difference spectra (Fig. 5A) show a more complex electronic signal than for WT, with increases in absorbance at 414, 426 and 440 nm, all of which have different amplitudes and decay to the ground state at varying rates. As expected, the ground‐state bleach remains at 393 nm, which corresponds to the loss of 5c‐NO. The increase in spectral complexity is possibly a result of R124A existing as a mixture of proximal and distal 5c‐NO species prior to excitation, consistent with the crystal structure (with occupancies of 0.7 for the heme with proximal NO and 0.3 for the heme with distal NO) 13. Furthermore, R124A appears to undergo a lower proportion of geminate recombination compared to WT (~ 15% of the initial signal amplitude remaining after 1 ns). This increase in residual signal after 1 ns may be due to enhanced solvent exposure at the heme proximal face when the large basic Arg124 is replaced by an Ala residue, suggesting a role for Arg124 in protecting against NO solvent escape. However, this spectral feature may instead be due to the existence of a mixture of proximally and distally bound NO in the case of R124A. In contrast to the TA findings, the TRIR data for R124A are comparable to those observed for WT (Fig. S11). The only exceptions are minimal shifts in peak positions from 1577 to 1573 and 1637 to 1632 cm^−1^. According to our peak assignments for WT (Table S1), the 1573 cm^−1^ shift is probably due to alterations in the heme–protein vibrational changes following excitation, possibly due to the mixture of proximally and distally bound 5c‐NO for R124A. This characteristic may also explain the peak shift at 1632 cm^−1^, which we assigned to loss of the His–Ala hydrogen bond, and may be affected vibrationally by the mixture of NO binding. As the overall R124A TRIR spectral profile resembles that of WT, this suggests that, following excitation, the 5c‐NO geminate recombination events are vibrationally similar, but have different electronic characteristics.

**Figure 6:**
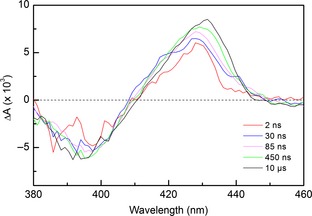
Absorption difference spectra for R124A between 2 ns and 10 μs. The 2 ns spectrum is similar in profile to the end of the ultrafast (fs–ns) experiments, with a signal bleach at 393 nm and corresponding positive transient absorption feature at 425 nm. An increase in absorbance with time is most clearly seen at 432 nm.

A significant proportion of the bound NO does not undergo rapid geminate recombination on the fs–ns time scale for the R124A variant, and additional spectral changes may be observed on the ns–μs time scale (Fig. 6). Over the subsequent 10 μs, there is a significant increase in amplitude at 432 nm, confirming that an additional process occurs for this variant. The absorbance band at 432 nm has been previously assigned to the 5c‐His species 19, and therefore suggests an increase in this population over μs time scales.

**Figure 7:**
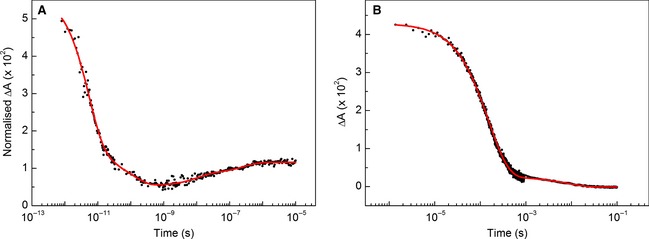
UV‐vis 432 nm signal decay following photolysis of 5c‐NO R124A between 1 ps and 100 ms. (A) Normalized absorbance measured from ps to μs fitted to the sum of five exponentials. The data acquired from the ps–ns and ns–μs experiments were normalized by their respective absorbance amplitudes at 1–3 ns. (B) Laser‐flash photolysis signal between 1 μs and 100 ms, fitted to the sum of two exponentials. Single‐wavelength data were acquired in order to achieve an optimal signal‐to‐noise ratio. The reduction in noise after ~ 1 ms is due to the different detection systems employed. The amplitude of ΔA between 1 and 10 μs (the overlapping region of the TA and flash‐photolysis experiments) is verified in the Data S1 section of the supplementary information.

In order to examine the TA data in its entirety, the data on the fs–μs time scales were combined, as described in Experimental procedures, to allow kinetic analysis of the whole dataset. Global analysis returned five spectral components instead of the three observed with WT, as detailed in Table 2. For illustrative purposes, the spectral change at 432 nm is shown in Fig. 7A, which shows the initial decrease in amplitude over ~ 1 ns, followed by a subsequent increase in absorbance over the following ~ 10 μs. The early lifetime values for R124A (τ_1_–τ_3_) are similar to those for WT, showing two fast phases (thought to be geminate recombination of NO; as for WT, the 1656 cm^−1^ feature of R124A relaxes to the ground state on the ~ 1 ps time scale) and an ~ 100 ps spectral feature representing His rebinding to the 4c‐heme. For R124A, the respective amplitudes of the individually fitted frequencies are larger than for WT, and therefore the global fit returned a lifetime with reduced error, and increased similarity to the predicted 100 ps value 19. The increase in τ_3_ signal amplitude for R124A may be due to additional His rebinding, and therefore, in the WT, Arg124 may act to repel His120 by virtue of charge and steric effects.

**Table 2:** Kinetic lifetime values from global fitting of TA and TRIR data for R124A across fs–μs (TA) and fs–ns (TRIR) time scales. These lifetime values were generated by global fitting to signal decays at the following frequencies: 393, 402, 416, 427, 431 and 440 nm for TA data, and 1575, 1597, 1626 and 1655 cm^−1^ for TRIR data. The τ_3_ value in the TRIR was poorly resolved due to the low number of data points acquired beyond 20 ps

	TA	TRIR
τ_1_	2.65 ± 0.24 ps	0.99 ± 0.12 ps
τ_2_	6.94 ± 0.24 ps	5.15 ± 0.17 ps
τ_3_	123 ± 7 ps	> 20 ps
τ_4_	17.5 ± 2.9 ns	–
τ_5_	302 ± 70 ns	–

**Scheme 2:**
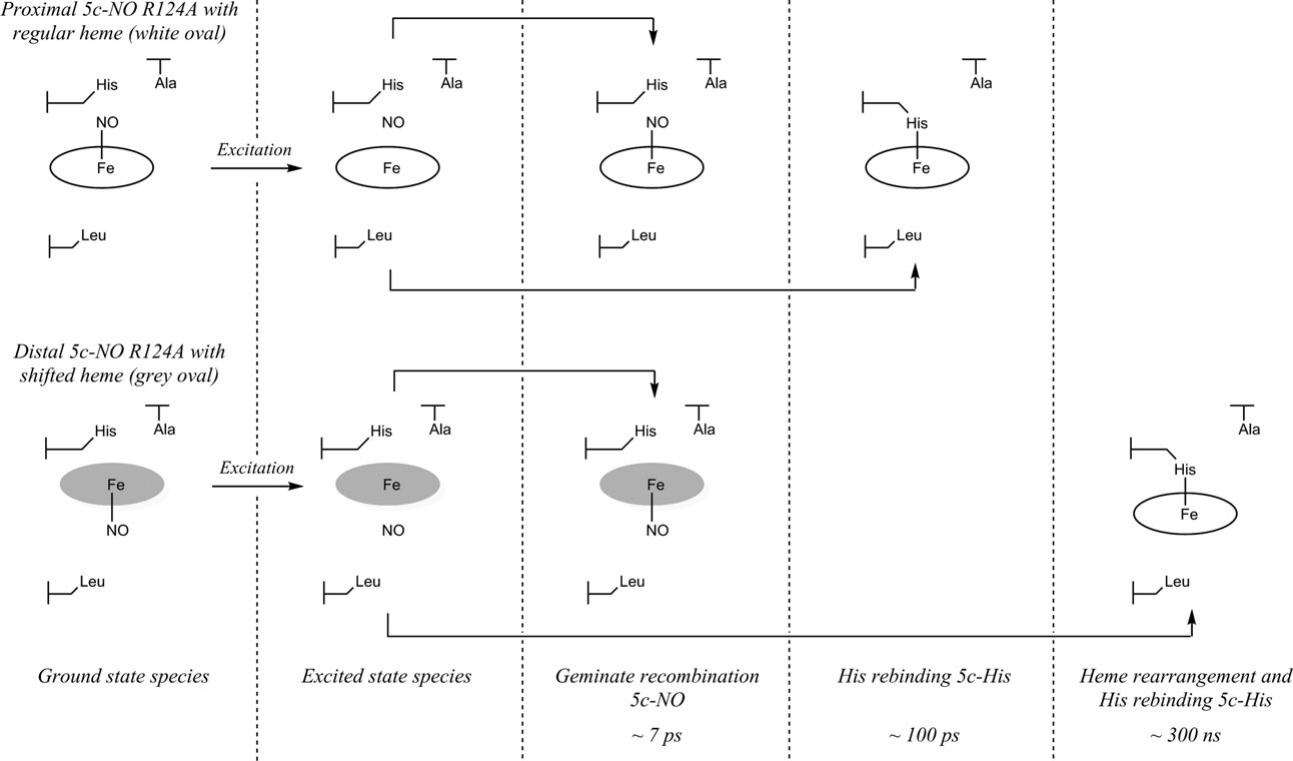
The proposed geminate recombination mechanism for R124A, which starts with a mixture of proximally and distally bound 5c‐NO, with the distally bound form having a shifted heme (indicated by grey shading). Upon excitation, both species undergo cleavage of Fe–NO, forming a 4c‐heme intermediate. Over the subsequent ~ 7 ps, geminate recombination occurs for a proportion of each species, which, according to the TA data, appears to be diminished with respect to the WT data, with both R124A species likely to contribute to greater NO solvent escape. The remaining proximally bound WT‐like species undergoes histidine rebinding over ~ 100 ps, forming a 5c‐His species, while the distally bound species must undergo heme rearrangement before histidine reattachment, which occurs over ~ 300 ns.

As R124A exists as a mixture of proximal and distal 5c‐NO, we suggest that the longer components correspond to reattachment of 5c‐His to the (originally) distal 5c‐NO species. This differs from the components present in the distal 6c‐NO complex produced at low NO concentrations in WT, where photo‐dissociation of NO leaves a 5c‐His species 17. In the case of R124A, there is a shifted heme with the proximal histidine stacked parallel to the heme plane. In order for the His to reattach, significant structural readjustment of the heme is necessary following NO dissociation, which probably occurs over longer time courses than the originally hypothesized kinetic trap mechanism for WT. We therefore infer that the longer components over ~ 300 ns correspond to this process, which eventually results in formation of a proximally bound 5c‐His species. The crystal structure of ferrous R124A supports this idea, as only 5c‐His is observed in the reduced form 13, analogous to the WT reduced structure. Accordingly, all of the R124A species that have undergone NO solvent escape form an identical 5c‐His structure after ~ 300 ns. The proposed mechanism for R124A photolysis on these time scales is outlined in Scheme 2. This hypothesis relies on the existence of a relatively long‐lived, highly reactive 4c‐heme species, and therefore awaits confirmation, for example by molecular dynamic simulation modeling.

A significant absorbance peak remains at 432 nm for the R124A variant after 10 μs, but subsequent laser‐flash photolysis experiments have shown that this decreases on the μs–s time scale (Fig. 7B). Moreover, analogous stopped‐flow UV‐vis measurements have shown that, in contrast to WT, the 6c‐NO distal species is not observed in the R124A variant. Instead, only the 395 nm 5c‐NO and 423/432 5c‐His species are observed, with a clear isosbestic point at 408 nm (Fig. S12). This has been ascribed to an increased *k*_6−5_ in the absence of the effects of an arginine at residue 124. A single exponential fit at increasing NO concentrations provides a *k*_on_ value of 6.42 ± 0.09 × 10^4^ m^−1^ s^−1^ (Fig. S13; reported literature value = 3.2 ± 0.1 × 10^4^ m^−1^ s^−1^) 13. In contrast to the stopped‐flow findings, the signal decay from the laser photoexcitation measurements in Fig. 7B fits to the sum of two exponentials and reveals a previously unreported fast phase on the ~ 100 μs time scale. To determine whether this was a second‐order process, laser‐flash photolysis measurements were repeated at a range of NO concentrations for the R124A variant, and showed that both *k*_1_ and *k*_2_ are dependent on NO concentration (Figs S14 and S15). *k*_2_ has a calculated *k*_on_ of 7.23 ± 0.92 × 10^4^ m^−1^ s^−1^, and is therefore likely to represent the same binding event previously reported in the stopped‐flow studies (formation of a 5c proximal NO species). However, *k*_1_ has a calculated *k*_on_ value of 3.50 ± 0.15 × 10^6^ m^−1^ s^−1^, which had not been observed in previous stopped‐flow experiments (the *k*_off_ value was negligible for both *k*_1_ and *k*_2_). However, at low concentrations of NO (0.1 mm), the signal decay of stopped‐flow R124A also fits to the sum of two exponentials, with a rate of decay similar, within an order of magnitude, to that for the laser‐flash photolysis experiments. Due to the time resolution of the stopped‐flow instrument (~ 1 ms), this fast phase fitted only a few data points, hence there is a large error in the rate determined. As this rebinding event from solvent is also NO‐dependent, it may correspond to formation of the distal 5c‐NO species (including formation of an initial 6c‐NO species, dissociation of histidine and collapse of the heme structure as an alternative pathway to that would yield proximal NO).

### Concluding remarks

The ability of cytochrome c' to bind NO and CO on opposite faces of the heme, and the availability of variants in which ligand binding is radically altered, renders it an excellent model system for understanding how precise structural arrangements modulate the affinity of the heme cofactor for these diatomic gases in hemoproteins. By using a variety of time‐resolved spectroscopic techniques, spanning time scales from femtoseconds to seconds, we have shown the importance of the heme pocket architecture in regulating the affinity for NO. Previous stopped‐flow, TA and crystallographic studies have indicated that cytochrome c' binds NO in a tightly regulated manner, with a number of implicated heme pocket residues 11. Our findings indicate that removal of a single proximal residue (R124) significantly affects the tightly regulated NO rebinding events upon excitation in WT. In the R124A variant of cytochrome c', there is a significantly different mechanism for heme–NO rebinding, with an increase in NO escape to solvent and an extension into the μs window, which was not observed for either the WT or L16A variant. This probably represents a readjustment of the heme cofactor for the distal 5c‐NO species and proximal rebinding of His120 to re‐form the starting 5c‐His species. In addition, there are two distinct, slower processes in R124A only, which probably correspond to heme collapse and formation of a distal 5c‐NO species (fast phase) and formation of a proximal 5c‐NO species (slow phase). These results indicate the complexity of NO rebinding in the R124A variant, and illustrate the crucial role of this residue in controlling the heme pocket reactivity towards NO.

In the wider context, the ability to monitor ligand photolysis in proteins from initial chemical events (fs) to the final equilibrium position (s) provides an exciting opportunity to monitor protein dynamics corresponding to ligand binding processes. Furthermore, the importance of the heme pocket architecture in modulating the control of heme–ligand reactivity has been emphasized, which may have significant implications for other gas‐binding proteins.

## Experimental procedures

### *Ax*Cytcp preparation

Wild‐type, R124A and L16A *Ax*Cytcp were over‐expressed and purified as previously described 12. WT and R124A samples were isolated in MES buffer, pH 6.0, in the ferric form, and purity was estimated by SDS/PAGE and UV‐vis spectroscopy, with the concentration being estimated using ε_400_ = 80 000 m^−1^ cm^−1^ 31. Samples were reduced using an excess of sodium dithionite (~ 10 mm) in an anaerobic glove box (Belle Technology, Weymouth, UK), and passed down a desalting column (Centri Pure P25; EMP Biotech, Berlin, Germany) equilibrated with anaerobic 50 mm N‐Cyclohexyl‐2‐aminoethanesulfonic acid (CHES) buffer, pH 8.9 (D_2_O for IR measurements adjusted to pD 8.9) to remove excess reductant. The concentration of the ferrous sample was estimated using ε_426_ = 97 000 m^−1^ cm^−1^ 31. The L16A variant was isolated with CO bound to the heme distal face as previously reported 12. This ligand was removed by incubating with an excess of potassium ferricyanide (500 mm) for 1 h at room temperature under anaerobic conditions. Excess oxidant was removed by passage down a desalting column equilibrated with anaerobic 50 mm MES buffer, pH 6.0. The sample was then reduced using an excess of sodium dithionite (~ 10 mm), and passed down a desalting column pre‐equilibrated with anaerobic 50 mm CHES buffer, pH 8.9 (D_2_O for IR measurements adjusted to pD 8.9). The concentration was estimated using ε_420_ = 80 000 m^−1^ cm^−1^.

### TA measurements

A Ti:sapphire amplifier (a hybrid Legend Elite‐F‐HE; Coherent Inc., Santa Clara, CA, USA) was pumped by a Q‐switched Nd:YLF laser (Evolution‐30; Positive Light Inc., Santa Clara, CA, USA) and seeded using a Ti:sapphire laser (Mai Tai; Spectra Physics, Santa Clara, CA, USA). The amplifier has an output wavelength of 800 nm, a 1 kHz repetition rate, and a 120 fs pulse duration. This beam is then split, with part of the output used to produce tuneable radiation in the range 250–1000 nm via a non‐collinear optical parametric amplifier (TOPAS‐White, Light Conversion Ltd., Vilnius, Lithuania). Another fraction of the amplifier output is used to pump Helios and Eos broadband pump‐probe transient absorption spectrometers (Ultrafast Systems LLC, Sarasota, FL, USA), with instrument response functions of ~ 170 fs and 500 ps, respectively. In all cases, samples were excited at 532 nm with 0.5–1 μJ power and a beam diameter of ~ 150 μm. Absorption changes were monitored between 350 and 700 nm at time delays between 100 fs and 3 ns after excitation for Helios experiments and between 0.5 ns and 400 μs for Eos experiments. Reduced samples were added to 2 mm path‐length quartz cuvettes and adjusted to a concentration such that the Soret band had an absorbance reading of ~ 0.7 (~ 44 μm). A Suba‐seal (Sigma‐Aldrich, St. Louis, MO, USA) septum was then attached to the cuvette entrance inside an anaerobic glovebox (Belle Technology), and NO gas was bubbled into the cuvette until the spectrum resembled the respective NO‐bound profile. Samples were stirred to avoid photobleaching.

### Flash photolysis measurements

Laser‐flash photolysis measurements were performed as described previously 32, using a 150 W xenon arc lamp probe that is pulsed for measurements > 1 ms and adjusted to the probing wavelength using an input monochromator. For saturated solutions (2 mm) of NO, samples were prepared by injecting NO gas into 1 cm path‐length quartz cuvettes sealed with Suba‐seal septums containing reduced *Ax*Cytcp under anaerobic conditions. The NO‐bound Soret absorbance reading was then adjusted to ~ 0.7 (~ 9 μm). An excitation wavelength of 532 nm was generated using the second harmonic of a Q‐switched Nd:YAG laser (Brilliant B; Quantel, Les Ulis, France) with a laser energy at the sample of 100 mJ and a beam diameter of ~ 1 cm. Five replicates were acquired per sample, and the temperature was maintained at 25 °C. The detection system for ms–s measurements utilized a photomultiplier tube (Applied Photophysics, Leatherhead, UK), whilst faster μs–ms measurements were acquired using an Infiniium oscilloscope (model number 54830B, Agilent technologies, Santa Clara, CA, USA) 32. For NO concentration‐dependence studies of the R124A variant, a saturated solution of NO was prepared by adding anaerobic 50 mm CHES buffer, pH 8.9, to a 50 mL conical flask, followed by attachment of a Suba‐seal septum. NO gas was then injected into the conical flask, replacing 25 mL of buffer, and mixed thoroughly. This NO solution was diluted appropriately by injection into a cuvette sealed with a Suba‐seal septum containing R124A and a varying volume of anaerobic buffer. These samples were analyzed shortly afterwards to prevent fluctuation in NO concentration, and seven replicates were used to calculate shot‐to‐shot error.

### Stopped‐flow UV‐vis spectroscopy

For stopped‐flow measurements, WT and R124A were prepared to a concentration of 12 μm (6 μm post‐mixing), while L16A was prepared to 4 μm (2 μm post‐mixing) due to its higher affinity for NO. For NO concentration‐dependence measurements, a stock NO solution (2 mm) was prepared in a 50 mL conical flask as for laser‐flash photolysis. Under anaerobic conditions, the NO stock solution was diluted appropriately to the desired concentrations with 50 mm CHES buffer, pH 8.9. Photodiode array and single‐wavelength absorption measurements were acquired using a TgK Scientific (Bradford‐on‐Avon, UK) stopped‐flow spectrometer housed inside an anaerobic glovebox (Belle Technology). Samples were rapidly mixed together to trigger NO binding, and six readings were performed to calculate shot‐to‐shot error.

### TRIR spectroscopy

Time‐resolved infrared measurements were performed using an ultrafast TRIR experimental set‐up described previously 33, with a 10 kHz repetition rate and 100 fs time resolution. *Ax*Cytcp samples were prepared to ferrous form at a concentration of ~ 2 mm, then either analyzed without NO bound or incubated with 3.5 mg spermine NONOate (Tocris Bioscience, Bristol, UK) for 4 h at room temperature to allow NO binding. Full NO binding to ferrous Cytcp samples was confirmed by UV‐vis spectroscopy (Fig. S1). Samples were then added to an anaerobic cell with CaF_2_ windows and a 75 μm spacer, which was rastered to avoid sample damage. For all samples, an excitation wavelength of 532 nm was used with 1 μJ pulse power and a beam diameter of ~ 150 μm, the polarisation was set at the magic angle with respect to the IR probe beam. Spectra were measured at time delays ranging between 500 fs and 1 ns. Difference spectra were generated relative to the ground state in the spectral window 1300–1800 cm^−1^. The spectra were measured using two 128‐pixel detectors with a spectral resolution of ~ 3 cm^−1^ per pixel. Pixel to wavenumber calibration was performed as described previously 34.

### Global fitting

Kinetic analyses were performed using origin 8.5 (Originlab Corp., Northampton, MA, USA) software. Five spectral positions of significant absorption change in the TA and TRIR were selected and fitted using shared lifetime values with the global fitting option. This returns a single lifetime value for each exponential, and a number of amplitude values for each of the respective frequencies. The sample concentration and pump power were similar in all the TA experiments performed; however, when combining the fs–ns and ns–μs datasets to span the full fs–μs time range, a small scaling factor (1.2) was applied to the ns–μs dataset to ensure a smooth match to the fs–ns dataset.

## Supplementary Material

**Fig. S1.** UV‐vis spectra for reduced and NO‐bound samples of WT, R124A and L16A.
**Fig. S2.** Infrared spectra for reduced and NO‐bound WT, R124A and L16A, plus difference spectra (NO‐bound minus reduced).
**Fig. S3.** Reduced WT TRIR difference spectra relative to the ground state between 1 and 100 ps after excitation.
**Fig. S4.** Comparison of 1 ps difference spectrum of WT with NO bound with the 1 ps difference spectrum of a WT reduced sample.
**Fig. S5.** Comparison of TRIR reduced samples for WT, R124A and L16A 1 ps spectra normalized to maximum and minimum amplitudes.
**Fig. S6.** WT TA absorption at five distinct wavelengths fit to the sum of three exponentials using shared lifetimes and a non‐linear least squared fitting model.
**Fig. S7.** Residual plots of 393 and 428 traces fit using the global fitting model shown in Fig. S6.
**Fig. S8.** 1656 cm^−1^ kinetic decay from WT TRIR data fit to the sum of two exponentials.
**Fig. S9.** Laser‐flash photolysis data at 396 nm for WT fit to the sum of two exponentials.
**Fig. S10.** Comparison of TRIR 1 ps difference spectra for WT and L16A.
**Fig. S11.** Direct comparison of TRIR WT and R124A 1 ps difference spectra.
**Fig. S12.** Photodiode array spectra of R124A versus 0.2 mm NO at time points between 1 and 500 ms post‐mixing.
**Fig. S13.** NO concentration‐dependence curve for R124A versus NO acquired by stopped‐flow UV‐vis.
**Fig. S14.** R124A laser‐flash photolysis concentration dependence curve for *k*_1_ rates against six NO concentrations.
**Fig. S15.** R124A laser‐flash photolysis concentration dependence curve for *k*_2_ rates against six NO concentrations.
**Table S1.** Assignment of TRIR difference spectra features for WT.
**Table S2.** The influence of NO and protein concentration on the exponential fit of L16A.
**Data S1.** Agreement of Δ*A* values between R124A TA and flash‐photolysis experiments.
