# Supplementary material for: Modulation of ligand–heme reactivity by binding pocket residues demonstrated in cytochrome c' over the femtosecond–second temporal range
Source: FEBS J. 2013 Oct 11;280(23):6070–82. doi: 10.1111/febs.12526 (PMC4163637; doi:10.1111/febs.12526)
Supplement: Supplementary file 1 — Fig. S1. UV‐vis spectra for reduced and NO‐bound samples of WT, R124A and L16A. Fig. S2. Infrared spectra for reduced and NO‐bound WT, R124A and L16A, plus difference spectra (NO‐bound minus reduced). Fig. S3. Reduced WT TRIR difference spectra relative to the ground state between 1 and 100 ps after excitation. Fig. S4. Comparison of 1 ps difference spectrum of WT with NO bound with the 1 ps difference spectrum of a WT reduced sample. Fig. S5. Comparison of TRIR reduced samples for WT, R124A and L16A 1 ps spectra normalized to maximum and minimum amplitudes. Fig. S6. WT TA absorption at five distinct wavelengths fit to the sum of three exponentials using shared lifetimes and a non‐linear least squared fitting model. Fig. S7. Residual plots of 393 and 428 traces fit using the global fitting model shown in Fig. S6. Fig. S8. 1656 cm−1 kinetic decay from WT TRIR data fit to the sum of two exponentials. Fig. S9. Laser‐flash photolysis data at 396 nm for WT fit to the sum of two exponentials. Fig. S10. Comparison of TRIR 1 ps difference spectra for WT and L16A. Fig. S11. Direct comparison of TRIR WT and R124A 1 ps difference spectra. Fig. S12. Photodiode array spectra of R124A versus 0.2 mm NO at time points between 1 and 500 ms post‐mixing. Fig. S13. NO concentration‐dependence curve for R124A versus NO acquired by stopped‐flow UV‐vis. Fig. S14. R124A laser‐flash photolysis concentration dependence curve for k1 rates against six NO concentrations. Fig. S15. R124A laser‐flash photolysis concentration dependence curve for k2 rates against six NO concentrations. Table S1. Assignment of TRIR difference spectra features for WT. Table S2. The influence of NO and protein concentration on the exponential fit of L16A. Data S1. Agreement of ΔA values between R124A TA and flash‐photolysis experiments. [file febs-280-6070-s1.zip › febs12526-sup-0001-FigS1-S15_TableS1-S2.pdf]

the FEBS Journal
WILEY Blackwell
www.febsjournal.org

Modulation of ligand–heme reactivity by binding pocket residues demonstrated in cytochrome c’ over the femtosecond–second temporal range

Henry J. Russell, Samantha J. O. Hardman, Derren J. Heyes,
Michael A. Hough, Gregory M. Greetham, Michael Towrie, Sam Hay and
Nigel S. Scrutton

DOI: 10.1111/febs.12526

SUPPLEMENTARY MATERIAL

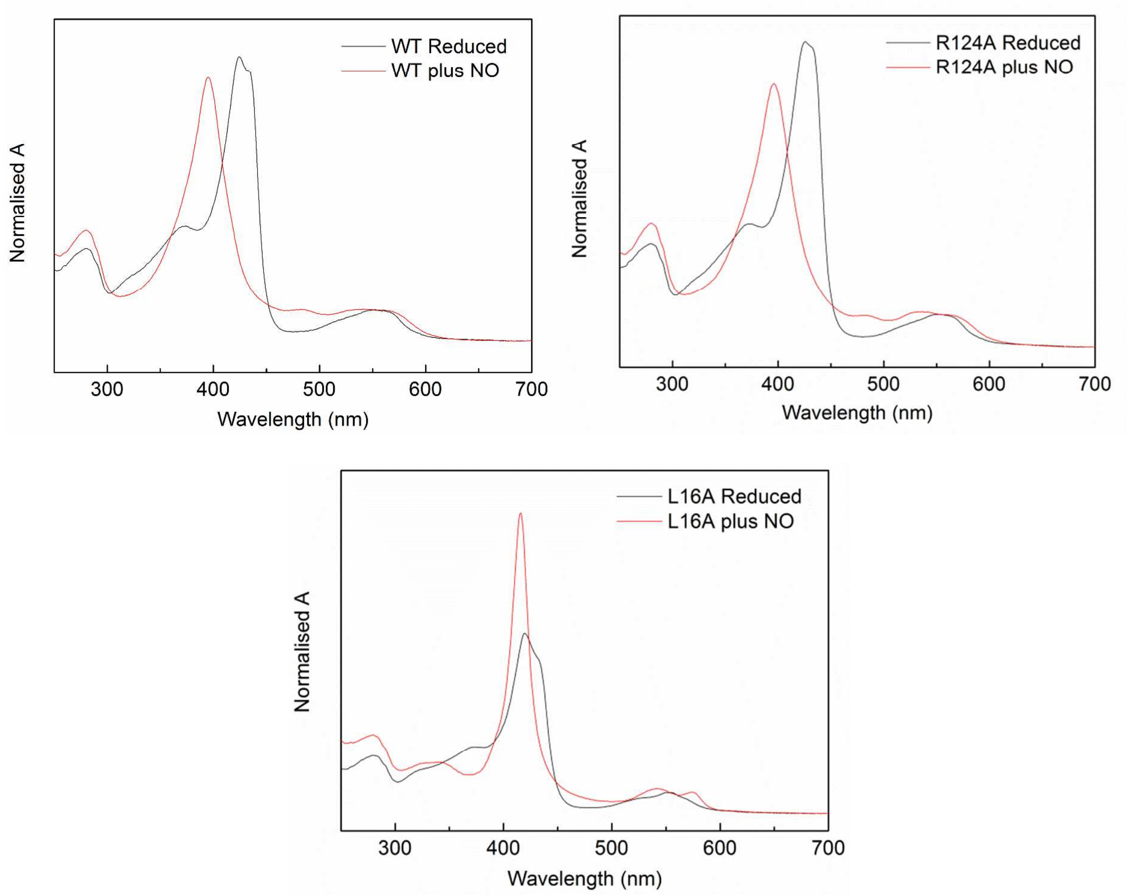

Figure S1. UV-vis spectra for reduced and NO-bound samples of WT, R124A and L16A normalised to A_{700}. When reduced, WT (top, left) exhibits a split Soret band with peak positions 424 and 432 nm which shifts to a single Soret upon NO-binding centred at 395 nm. R124A (top, right) has the same peak positions as the wild-type, with the only difference being the reduction in difference between peak intensities in the reduced 424/432 split Soret. L16A (bottom, middle) also has a split Soret in the reduced form with peak positions 420 and 432 nm which upon binding NO exhibits a sharp increase at 416 nm, corresponding to the formation of a 6c-NO species.

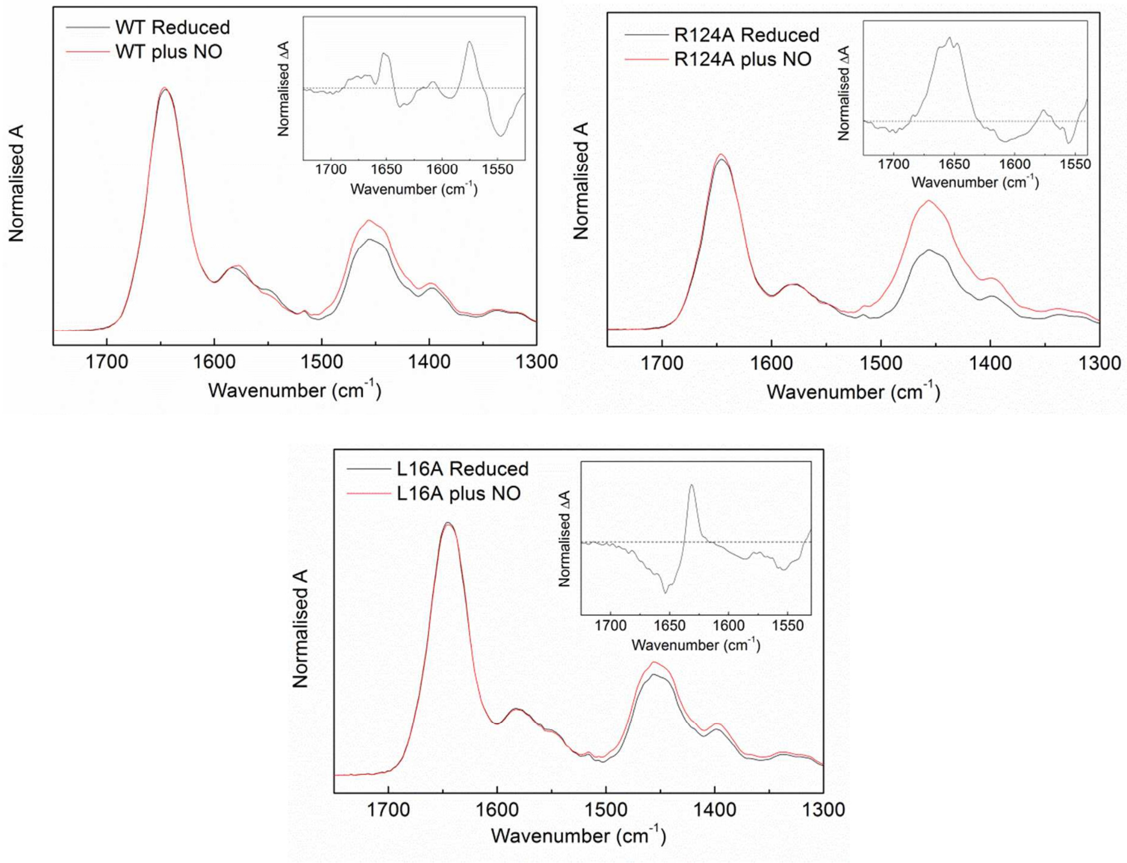

Figure S2. Infrared spectra for reduced (black traces) and NO-bound (red traces) WT, R124A and L16A, with difference spectra (NO-bound minus reduced) inset. All spectra were normalised to frequencies of no signal change. In all cases the spectra illustrate characteristic protein spectral structure, with an amide I band centred at 1645 cm^{-1} and amide II centred at 1455 cm^{-1}. The amide II band appears to broadly increase in all cases upon addition of NO, while the amide I stretch shows more subtle changes. For WT (top, left) there are a number of increases which complement existing IR assignments for NO binding to cytochrome c' [1]. R124A (top, right) illustrates similar difference spectra to the wild-type, the main difference being a more predominant IR increase at 1655 cm^{-1}. L16A (middle, bottom) returned difference spectra with an increase at 1630 cm^{-1} and corresponding decrease at 1655 cm^{-1} reflecting the 6-coordinate binding event for this variant.

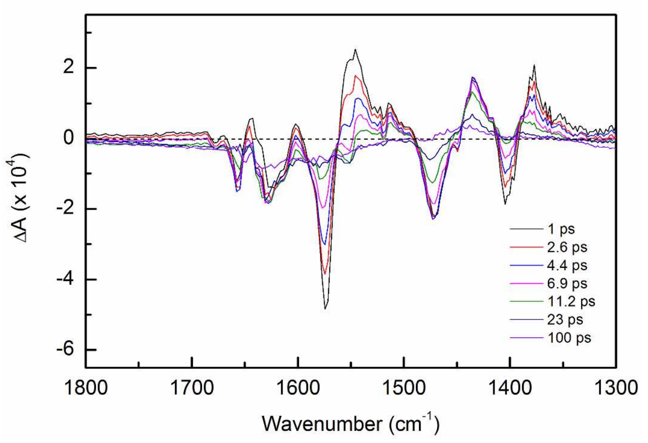

Figure S3. Reduced WT TRIR difference spectra relative to the ground state between 1-100 ps after excitation. This illustrates signal bleaches at 1575, 1471 and 1405 cm^{-1}, and transient features at 1377, 1436 and 1546 cm^{-1}, with a small but markedly reduced signal bleach at 1656 cm^{-1} which corresponds to a lack of 5c-NO in this species.

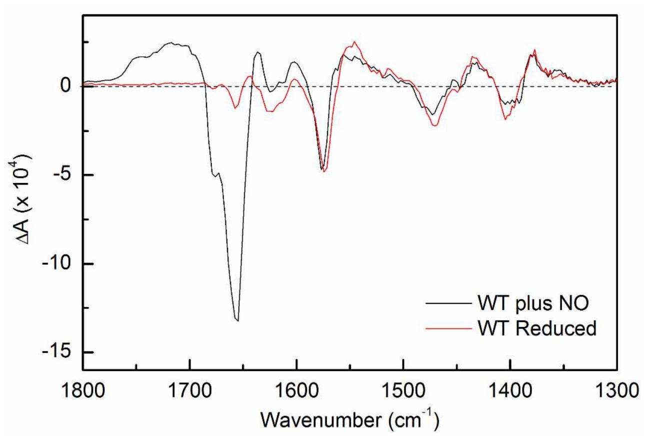

Figure S4. Comparison of 1 ps difference spectrum of WT with NO bound (black trace) with the 1 ps difference spectrum of a WT reduced sample (red trace). In the 1300-1600 cm^{-1} region the majority of signals are identical in position and amplitude. The major differences are the size of the 1655 cm^{-1} bleach and 1676 cm^{-1} shoulder, in addition to transient features centred at 1717 and 1637 cm^{-1}.

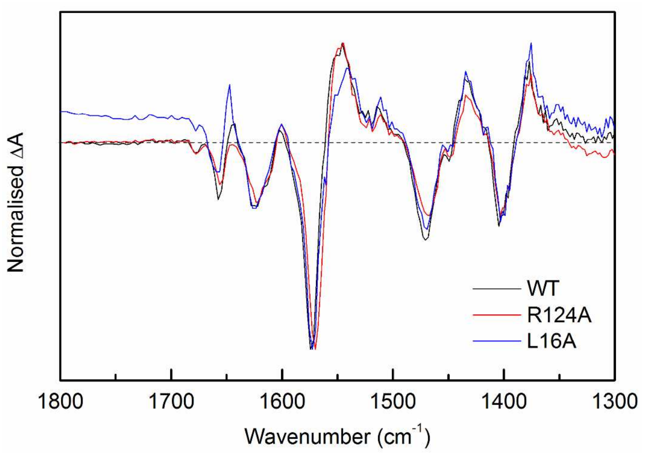

Figure S5. Comparison of TRIR reduced samples for WT, R124A and L16A 1 ps spectra normalised to maximum and minimum amplitudes. These spectra exhibit broadly identical spectral features, the most important of which being the presence of the 1575 cm^{-1} bleach, which suggests this is unlikely to correspond to the proximal arginine residue, or the histidine residue (this is also present in the NO bound spectra – Figure S2).

Table S1. Assignment of TRIR difference spectra features for WT.

TRIR Absorption Band (cm^{-1})	Assignment	IR Absorption Frequency in D_2O (cm^{-1})	Reference
1710-1750	Asp121, *v*(C=O)	1716-1775	[2]
1656	5c-NO-Heme	1656	[1]
1634	His, *v*(C=C)	1623	[3]
1300-1600	Heme vibrational modes after excitation	1300-1600	[4]

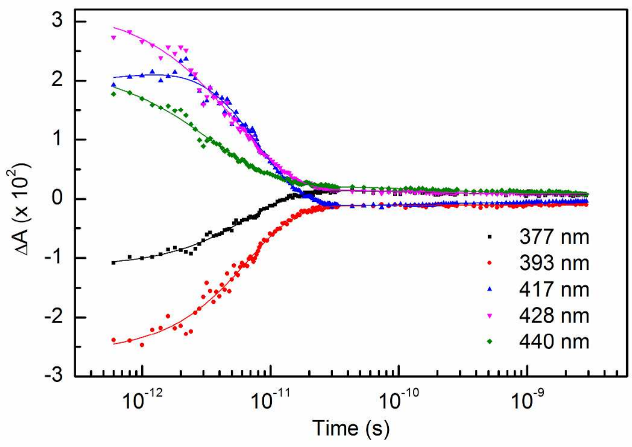

Figure S6. WT TA absorption at five distinct wavelengths fit to the sum of three exponentials using shared lifetimes and a non-linear least squared fitting model.

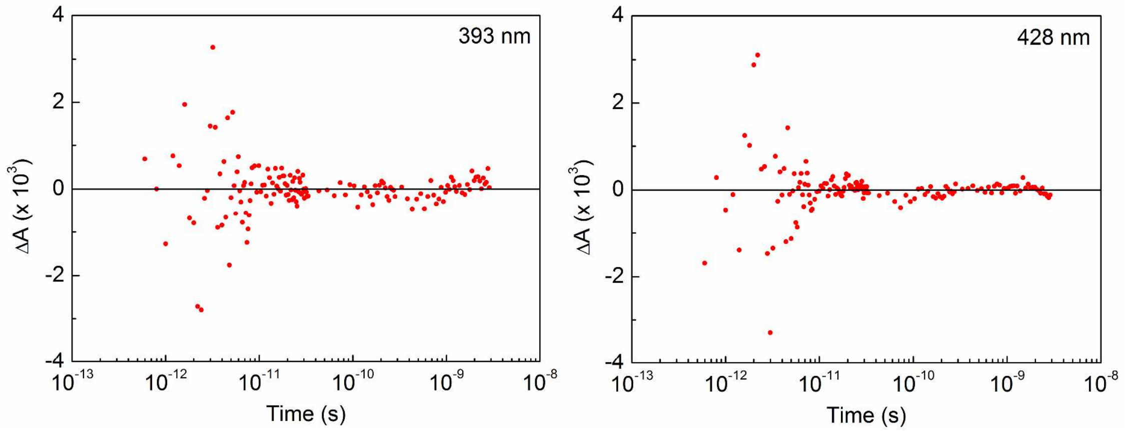

Figure S7. Residual plots of 393 and 428 traces fit using the global fitting model shown in Figure S6.

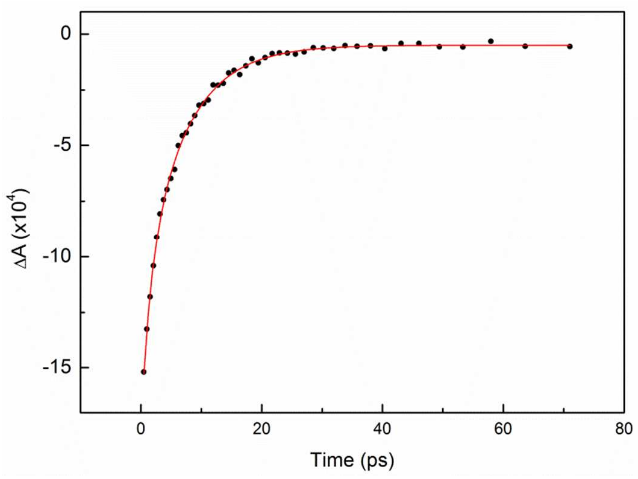

Figure S8. 1656 cm^{-1} kinetic decay from WT TRIR data fit to the sum of two exponentials. These kinetics were comparable to those reported by global fitting in Table 1, and the signal decay at time points < 1 ps indicate that the calculated τ_1 value by global fitting (2.19 ± 0.18 ps for TA; 1.31 ± 0.16 ps for TRIR) reports on NO geminate rebinding.

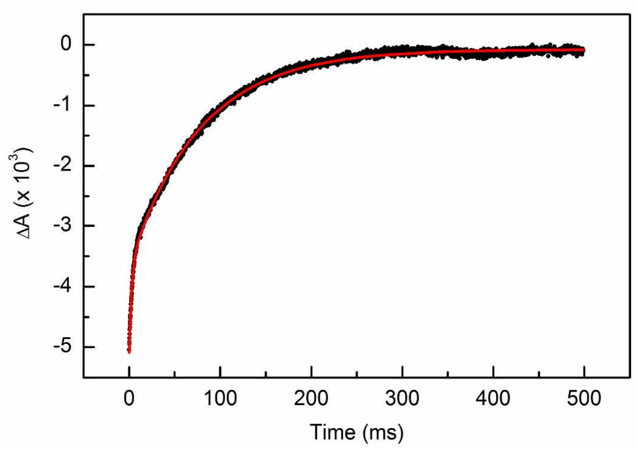

Figure S9. Laser-flash photolysis data at 396 nm for WT fit to the sum of two exponentials.

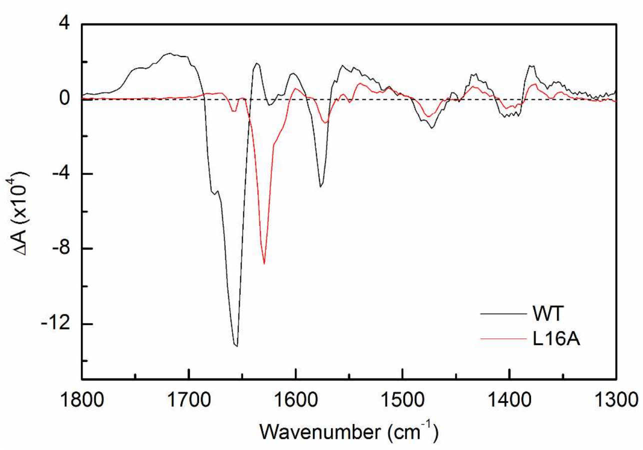

Figure S10. Comparison of TRIR 1 ps difference spectra for WT and L16A. Broadly identical spectral features are observed from 1300-1600 cm^{-1}, which most likely correspond to heme vibrations. The major spectral differences are the shift in bleach from 1656 to 1629 cm^{-1} which correlates to 5c-NO and 6c-NO binding respectively, and the loss of transient signal at 1717 cm^{-1}.

Table S2. The influence of NO and protein concentration on the exponential fit of L16A. Despite these rates reporting on geminate recombination processes, it appears they are dependent on the NO:protein concentration ratio, particularly for τ_1. It is likely that τ_1 corresponds to heme vibrational relaxation events; however, for this to be markedly affected by the concentration of NO and L16A is unusual and may require further experimentation for elucidation.

**Experiment**	**[L16A]**	**[NO]**	**τ_1**	**τ_2**
TA	50 µM	100 % (2 mM)	1.21 ± 0.21 ps	15.54 ± 0.89 ps
TA	50 µM	Determined by NONOate – low	3.85 ± 0.17 ps	18.45 ± 0.42 ps
TRIR	2 mM	Determined by NONOate – low	5.85 ± 0.59 ps	24.6 ± 2.2 ps

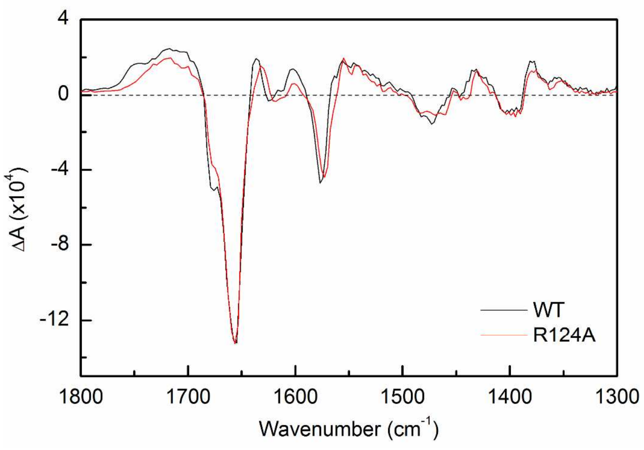

Figure S11. Direct comparison of TRIR WT and R124A 1 ps difference spectra. These spectra shown the same overall spectral structure, with the most noteworthy differences being a shifts in peak positions from 1577 to 1573 cm^{-1}, and from 1637 to 1632 cm^{-1}.

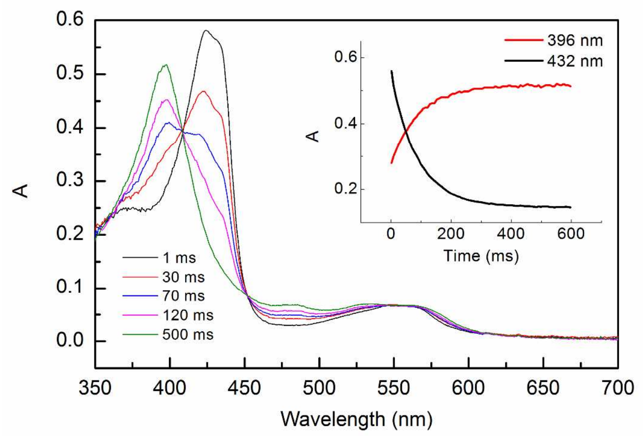

Figure S12. Photodiode array spectra of R124A vs 0.2 mM NO at time points between 1 – 500 ms post-mixing. This shows a decrease in amplitude in the 425/432 nm reduced peaks and an increase at 396 nm (NO bound). The respective decrease and increase in signal at 432 and 396 are illustrated inset.

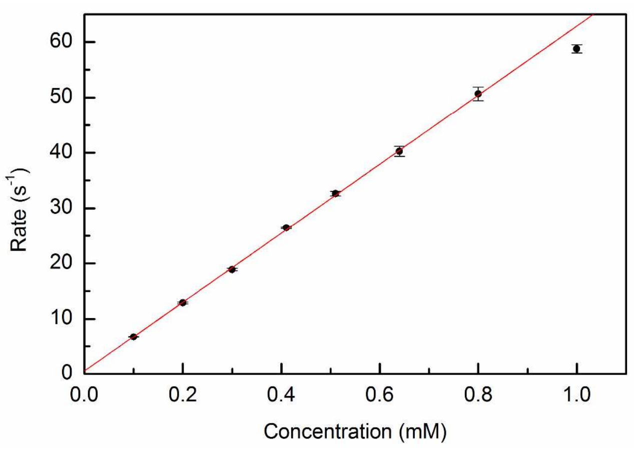

Figure S13. NO concentration-dependence curve for R124A vs NO acquired by stopped-flow UV-vis. The rates were calculated by fitting of the 432 nm trace at various NO concentrations.

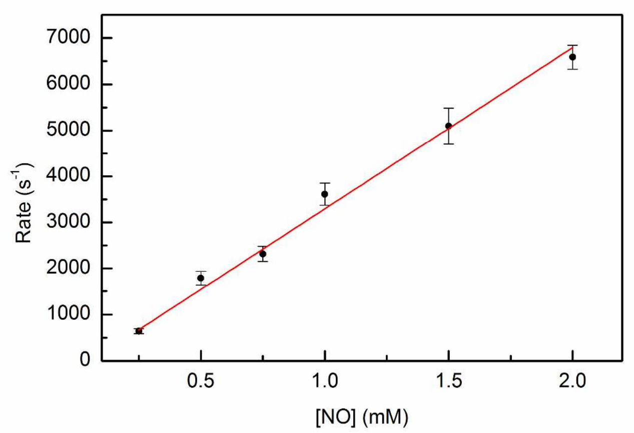

Figure S14. R124A laser-flash photolysis concentration dependence curve for k_1 rates against six different NO concentrations. The plot shows a linear dependence, with k_{on} value of (3.50 ± 0.15) × 10^6 $M^{-1}s^{-1}$.

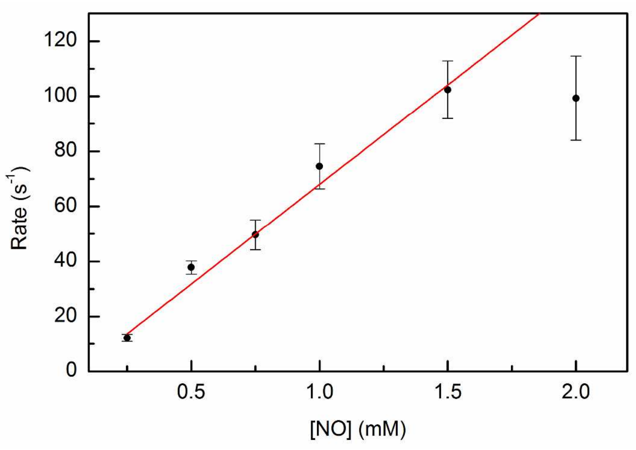

Figure S15. R124A laser-flash photolysis concentration dependence curve for k_2 rates against six differing NO concentrations. The plot shows a linear dependence, with k_{on} value of (7.23 ± 0.92) × 10^4 $M^{-1}s^{-1}$. The 2 mM sample returned a lower rate than expected from the linear regression, which could be due to the different sample preparation method used for this sample (see Experimental Procedures section).

Agreement of ΔA values between R124A TA and flash-photolysis experiments

The concentration of the R124A samples used for the TA and laser flash photolysis experiments were ~ 44 µM and ~ 9 µM respectively. These were set so that the Soret band (395 nm) absorption was 0.7 in the probe beam path (0.2 and 1 cm, respectively). The cuvette pathlength for the pump beam differed between the systems (2 mm and 4 mm, respectively), so the absorption at the Soret band in the pump beam was 0.088 and 0.035, respectively. The transient absorption experiments were performed with a power density of ~ 5.7 mJ/cm^2 (1 µJ, 150 µm beam diameter), and the flash photolysis experiments with a power density of ~ 127 mJ/cm^2 (100 mJ, 1 cm beam diameter). Between 1 and 10 µs the change in absorbance values were ~ 0.011 and ~ 0.041 for the TA and the flash photolysis experiments, respectively.

After correction for the different absorption cross sections in the two experiments, the TA and laser-flash photolysis values for ΔA/A are: 0.13 and 1.17, respectively. If this value is then divided by the power density, the resulting values are 0.022 and 0.009. In a perfect system these values should be the same, however factors such as pump/probe beam overlap, and the differing spectral bandwidth of the pump beams (~ 40 nm and > 1 nm, respectively) will affect the values, so we feel that the values are exhibit enough similarity to show reasonable correlation between the systems.

REFERENCES

1. George SJ, Andrew CR, Lawson DM, Thorneley RN & Eady RR (2001) Stopped-flow Infrared Spectroscopy Reveals a Six-Coordinate Intermediate in the Formation of the Proximally Bound Five-Coordinate NO adduct of Cytochrome c'. *J Am Chem Soc* **123**, 9683-9684.
2. Chirgadze YN, Fedorov OV & Trushina NP (1975) Estimation of amino acid residue side-chain absorption in the infrared spectra of protein solutions in heavy water. *Biopolymers* **14**, 679 - 694.
3. Barth A (2000) The infrared absorption of amino acid side chains. *Prog Biophys Mol Biol* **74**, 141-173.
4. Hellwig P, Grzybek S, Behr J, Ludwig B, Michel H & Mantele W (1999) Electrochemical and Ultraviolet/Visible/Infrared Spectroscopic Analysis of Heme a and a3 Redox Reactions in Cytochrome c Oxidase from Paracoccus denitrificans: Separation of Heme a and a3 Contributions and Assignment of Vibrational Modes. *Biochemistry* **38**, 1685-1694.
